# Association between Dietary Inflammatory Index and Gastric Adenocarcinoma: A Multicenter Case-Control Study in Brazil

**DOI:** 10.3390/nu15132867

**Published:** 2023-06-24

**Authors:** Alex Richard Costa Silva, Valdete Regina Guandalini, Taísa Sabrina Silva Pereira, Longgang Zhao, Michael D. Wirth, James R. Hébert, Gisele Aparecida Fernandes, Paulo Pimentel de Assumpção, Mônica Santiago Barbosa, Maria Paula Curado

**Affiliations:** 1 Postgraduate Program in Oncology, A.C. Camargo Cancer Center, São Paulo 01509-900, Brazil; alex.richard@accamargo.org.br (A.R.C.S.); gisele.fernandes@accamargo.org.br (G.A.F.); 2 Postgraduate Program in Nutrition and Health, Health Sciences Center, Federal University of Espírito Santo, Vitória 29047-105, Brazil; valdete.guandalini@ufes.br; 3 Department of Integrated Education, Health Sciences Center, Federal University of Espírito Santo, Vitória 29047-105, Brazil; 4 Department of Health Science, University of the Americas Puebla, San Andres Cholula 72810, Mexico; taisa.sabrina@hotmail.com; 5 Department of Epidemiology and Biostatistics, Arnold School of Public Health, University of South Carolina, Columbia, SC 29208, USA; lz7@email.sc.edu (L.Z.); wirthm@email.sc.edu (M.D.W.); jhebert@mailbox.sc.edu (J.R.H.); 6 Department of Nutrition, Connecting Health Innovations LLC, Columbia, SC 29208, USA; 7 College of Nursing, University of South Carolina, Columbia, SC 29208, USA; 8 Group of Epidemiology and Statistics on Cancer, International Research Center, A.C. Camargo Cancer Center, São Paulo 01508-010, Brazil; 9 Oncology Research Center, Federal University of Pará, Belém 66073-005, Brazil; assumpcaopp@gmail.com; 10 Institute of Tropical Pathology and Public Health, Federal University of Goiás, Goiânia 74605-050, Brazil; santiago@ufg.br

**Keywords:** gastric cancer, energy-adjusted dietary inflammatory index, pro-inflammatory diet, Brazilian population, Latin American population, endoscopy, risk factor

## Abstract

Background: Few studies have evaluated the association between diet-related inflammation and gastric adenocarcinoma (GA) and evidence is scarce in Brazil. This study evaluated the association between a pro-inflammatory diet and GA. Methods: A multicenter case–control study was conducted in Brazil. A total of 1645 participants—492 cases, 377 endoscopy controls, and 776 hospital controls—were included. Energy-adjusted Dietary Inflammatory Index (E-DII^TM^) scores were derived from a validated food frequency questionnaire. We used binary and multinomial logistic regression models for the analysis of total GA, and its subtypes (cardia and non-cardia, intestinal, and diffuse histological subtypes). Results: In cases versus endoscopy controls, a pro-inflammatory diet, estimated by higher E-DII scores, was associated with a higher risk GA (OR_Q4vsQ1_: 2.60, 1.16–5.70), of non-cardia GA (OR: 2.90, 1.06–7.82), and diffuse subtype (OR: 3.93, 1.59–9.70). In cases versus hospital controls, higher E-DII scores were associated with a higher risk of GA (OR: 2.70, 1.60–4.54), of cardia GA (OR: 3.31, 1.32–8.24), non-cardia GA (OR: 2.97, 1.64–5.39), and both intestinal (OR: 2.82, 1.38–5.74) and diffuse GA (OR: 2.50, 1.54–5.11) subtypes. Conclusions: This study provides evidence that a pro-inflammatory diet is associated with an increased risk of GA in Brazil. E-DII requires the inclusion of sodium due to its importance in carcinogenesis.

## 1. Introduction

Gastric cancer (GC) was the fifth most common cancer and ranked fourth in cancer deaths throughout the globe in 2020, with estimates of 1.1 million new cases and 770,000 deaths [1]. In Brazil, the estimated incidence for the three-year period 2023–2025 is 21,480 new cases, which will make it the fifth most frequently diagnosed cancer in Brazil [2]. In 2020, there were 13,850 recorded deaths from GC in Brazil, comprising 6.1% of all cancer deaths and ranking fifth among leading causes of cancer death, of which 63% of deaths were among men [2].

The most frequent histological type of GC is gastric adenocarcinoma (GA), which accounts for 90% of all cases [3]. *Helicobacter pylori (H. pylori)* infection is the main risk factor for non-cardia GA; it accounts for 90% of cases and was identified as a Group I carcinogen by the International Agency for Research on Cancer in 1994, which reconfirmed this classification in 2009 [4,5]. Diet is an associated factor that can act by both potentiating and inhibiting the inflammatory process caused by the infection. In addition to H. pylori infection, prolonged exposure to various dietary factors that are considered aggressive to the gastric mucosa (e.g., alcohol, salt, smoked foods, processed meats, etc.) can lead to inflammation and premalignant lesions [4,6].

Diet is a key modifiable target for reducing the risk of chronic non-communicable diseases such as cancer. Thus, dietary factors remain one of the main drivers of the global burden of these diseases. Diet can affect risk through multiple mechanisms of action, including modulation of the gut microbiome, oxidative stress, and energy balance. Potential pro- or anti-inflammatory properties of dietary patterns, in addition to individual dietary components, underlie all these mechanisms [7,8,9]. Most studies on diet and GA have evaluated a single nutrient or a group of isolated foods, which may not reflect the full effect of diet-related inflammation associated with cancer, as individuals consume combinations of foods and nutrients. The United States (US) 2020–2025 Dietary Guidelines emphasizes the value of focusing on overall dietary patterns instead of looking exclusively at a single dietary component [10]. The concept of a “pro-inflammatory diet” has thus emerged, having been defined as foods and nutrients that can stimulate the release of pro-inflammatory mediators, contributing to or exacerbating chronic inflammation [7,8,11]. 

To assess the inflammatory potential of a diet, the most widely used tool is the Dietary Inflammatory Index (DII^®^), which was specifically designed to assess and provide quantitative information about the inflammatory potential of an individual’s diet [7]. Recent meta-analyses have shown an association between DII and GA in European and Asian populations, supporting the hypothesis that pro-inflammatory diets increase the risk of GA [12,13]. The energy-adjusted DII (E-DII^TM^), which was developed to account for differences in energy intake that could influence inflammation, also has been evaluated in different types of cancer, including prostate [14], breast [15], and glioma [16], and a more pro-inflammatory diet has been found to be associated with a higher risk of developing these cancers.

While some studies have investigated the link between the inflammatory potential of diet and GA, there are relatively few studies focusing on Latin American populations, and even fewer related to the Brazilian population. Given the mounting evidence of the role played by diet in the regulation of inflammation and carcinogenesis, there is a need for further research in this area. Therefore, the objective of this study is to investigate the association between pro-inflammatory diets and GA in a multicenter case–control study conducted in Brazil.

## 2. Materials and Methods

### 2.1. Study Population and Design

GE4GAC-Brazil is a large, hospital-based, multicenter case–control study carried out in five capitals of different Brazilian regions [17]. Briefly, all cases were diagnosed with GA, confirmed by histology and coded according to the International Classification of Diseases in Oncology (C16). Two control groups were used. Control I group included participants with gastric complaints who underwent endoscopy, with a negative diagnosis for GC or a premalignant lesion. Control II group was composed of hospital participants without a diagnosis of cancer or gastric complaints and who were recruited from non-oncology clinics and from Cancer Prevention Programs. The exclusion criteria for participants were previous malignancy, except for non-melanoma skin cancer; participants with impaired mobility due to illness or mental and cognitive conditions that prevented them from understanding the questions asked by the interviewers; and cases that had been diagnosed with GA more than two years prior to the interview or those with advanced cancer (i.e., terminal stage with no feasible chance of survival). The study was approved by the Committee on Ethics in Human Research of Antônio Prudente Foundation—A.C. Camargo Cancer Center, as well as the local ethics committees of the study centers, under registration on the Brazil platform linked to the National Health Council of Brazil (grant nº 4708881–February 2016, registration CAAE: 53166915.9.1001.5432). All participants provided written informed consent for data collection and storage.

This study used a database from three Brazilian capitals with complete data collection, São Paulo (São Paulo state; southeastern region/metropolitan), Belém (Pará state; northern region/Amazon Rainforest), and Goiânia (Goiás state; central-western region/agrobusiness). Recruitment was conducted from April 2016 to September 2022. Controls were matched on sex and age (i.e., ±5 years in the range of 18 to 75 years). In the central-western region, pairing was not performed. All participants provided written informed consent for data collection and storage.

**Figure 1:**
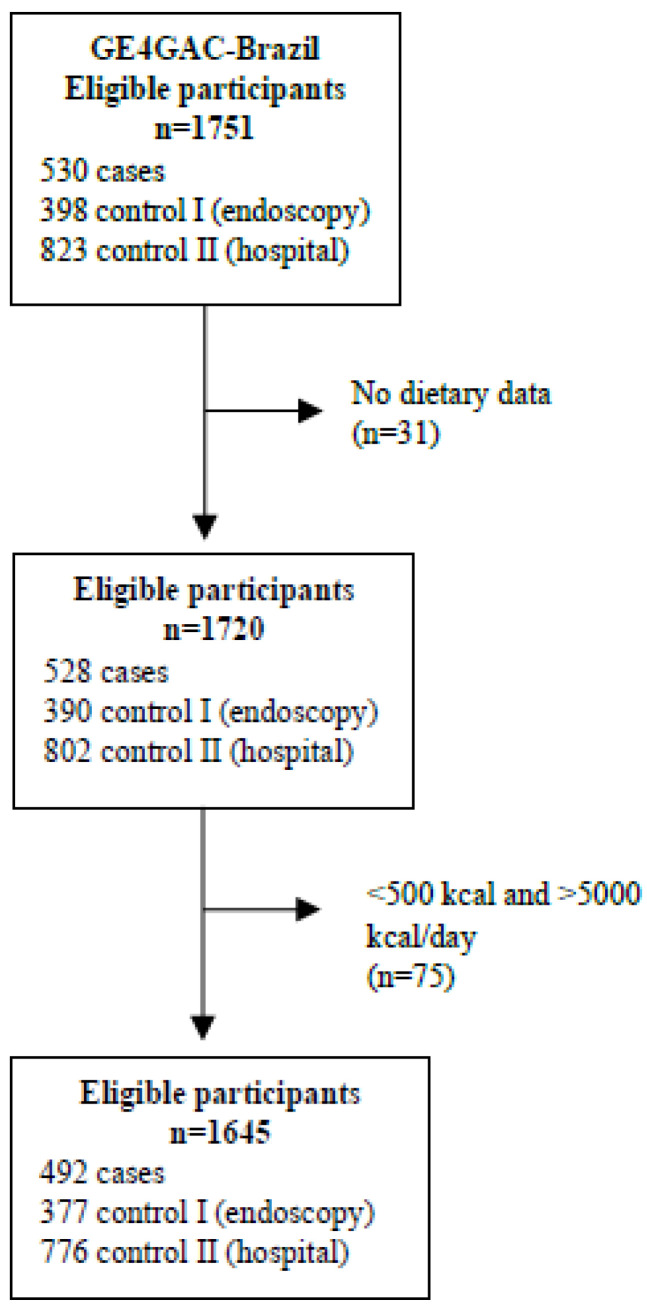
Flow chart of study population selection.

Participants who had an implausible energy intake of <500 and >5000 kcal per day were excluded from this study. A total of 1645 participants: 492 cases, 377 control I, and 776 control II, were included in the study (Figure 1). 

### 2.2. Data Collection

Face-to-face interviews were conducted at baseline by trained personnel using an online structured epidemiological questionnaire on the REDCap^®^ platform. Anonymized data were recorded on the platform at participating centers. We collected data on sex, age, marital status, schooling, self-reported ethnicity, and lifestyle habits (i.e., alcohol consumption and tobacco smoking). To calculate alcohol consumption (g/day), we used a previously reported method [18,19], while tobacco smoking was calculated in packs/year [19,20]. Information was collected on the presence of comorbidities, such as peptic ulcer and diabetes mellitus, that are associated with GA [19,21,22], and clinical data/family history of cancer in first-degree relatives. Information about the use of drugs aimed at controlling gastric acidity, treating gastroesophageal reflux, such as proton pump inhibitors (PPIs) and H2-receptor antagonists (H2Ras), antacids, aspirin, and other non-steroidal anti-inflammatory drugs (NSAIDs) were collected [23,24,25]. Additionally, in accordance with the guidelines of the World Health Organization (WHO), we measured weight (kg) and height (m) for the calculation of body mass index (BMI, kg/m^2^), and BMI classification (underweight/malnutrition: <18.5 (adults) and <21.9 (older adults); normal weight: 18.5 - < 25 (adults) and 22–27 (older adults); overweight: 25 - < 30 and 27.1 - < 30 (older adults); or obesity: ≥30 (adults and older adults) [26,27].

For cases, clinical information on anatomical location (cardia or non-cardia), tumor histological subtype (diffuse, intestinal, or mixed) [28], and status of *H. pylori* infection (positive or negative) were collected from medical records, the latter also having been collected for control I individuals based on gastric endoscopy reports. 

### 2.3. Dietary Assessment

An adapted food frequency questionnaire (FFQ) applied here includes >120 food items previously validated for a population with cancer [29]. Moreover, typical foods and preparations from the three Brazilian capitals in this study were added [30]. The FFQ asked about the food consumption patterns of the cases and both control groups in the prior twelve months. Each item was collected based on the frequency and portion size: (1) frequency of food consumption—ranging from 1 to 10 times daily, weekly, monthly, or yearly; and (2) size of the portion ingested—small, medium, or large (each food had its portion in grams and its equivalent in slices, spoons, and/or cups/glasses). Consumption of each item was calculated in grams per day. In addition, information on the consumption of nutritional supplements (yes/no) was collected.

Nutrient intake by cases and controls was determined using the Nutrition Data System for Research software—NDSR^®^ version 2021 (Minneapolis, MN, USA). To calculate the consumption of specific foods and preparations for the Brazilian regions evaluated here, we used the Brazilian Food Composition Table (TBCA) version 7.2 available at <http://www.fcf.usp.br/tbca> accessed on 2 January 2023 [31].

### 2.4. Assessing Dietary Inflammatory Potential

The inflammatory potential of the diet was calculated based on the DII, which was developed to quantify the inflammatory potential of individuals’ diets on a scale from maximally anti-inflammatory (most negative score) to maximally pro-inflammatory (most positive score). The development of the DII has been described in detail elsewhere [7]. Briefly, the DII scoring algorithm was based on a careful review of the literature through which 1943 articles identified 45 food parameters (i.e., macronutrients including specific categories of fatty acids, carbohydrate, and proteins; macronutrients including vitamins and minerals; flavonoids; and whole food items including herbs and spices) as having sufficiently robust literature in relation to six inflammatory biomarkers—i.e., interleukins (IL)-1b,-4,-6,-10, tumor-necrosis factor-alpha (TNF-α), and C-reactive protein (CRP). Self-report values for 30 of these food parameters were available from the FFQ. These were translated into z-scores using a global comparative database consisting of data from 11 countries by subtracting the mean of the global database from the individual’s self-report value and then dividing by the standard deviation. These scores were then converted to proportions (i.e., with values ranging from 0 to 1) and centered on zero by doubling each and subtracting 1. These centered proportions were then multiplied by their respective coefficients (overall food parameter-specific inflammatory effect scores) to obtain DII scores for each food parameter. These were summed to obtain the overall DII score. Energy-adjusted DII (E-DII^TM^) scores were calculated using the density approach by calculating DII per 1000 kcal consumption. This employed the same procedure for scoring but relies on an energy-adjusted global comparison database [32,33]. These DII and E-DII scores have a potential range from approximately −9 to +8; i.e., from minimally to maximally pro-inflammatory, respectively. The DII and E-DII are scored similarly and scaled identically; so, the scores are comparable across studies [33]. 

For this study, 30 of the 45 food parameters were used to calculate an individual’s overall DII score, including energy, carbohydrates, proteins, total fats, cholesterol, saturated fatty acids (SFAs), monounsaturated fatty acids (MUFAs), polyunsaturated fatty acids (PUFAs), trans fatty acids (TFAs), fiber, vitamins A, C, D, E, thiamin, riboflavin, niacin, vitamin B6, vitamin B12, magnesium, iron, selenium, zinc, caffeine, β-carotene, folic acid, omega-3, omega-6, onion, and pepper. For the E-DII, we used a density method to adjust for total energy intake for all dietary components; so, 29 parameters were used for computation. In this study, nutritional supplements were not included in the DII or E-DII calculation. The decision to use the E-DII was based on model goodness of fit analyzed by Hosmer–Lemeshow goodness-of-fit test.

### 2.5. Statistical Analyses

Continuous variables were expressed as median and percentiles (P_25_, P_75_). Categorical variables were expressed in absolute (n) and relative (%) frequencies. The Kolmogorov–Smirnov test was applied to verify the normality of continuous variables and differences were evaluated using the Kruskal–Wallis test with Dunn–Bonferroni post hoc test. For categorical variables, the Pearson χ^2^ or Fisher’s Exact tests was applied. Quartiles were determined based on the E-DII scores of the controls (control I and control II) separately; quartile 1 included participants with the lowest dietary inflammatory potential, quartiles 2 and 3 are intermediate, and quartile 4 included participants with the highest dietary inflammatory potential. 

The odds ratios (ORs) and respective 95% confidence intervals (CI) for the association between E-DII and GA in cases and controls were estimated by binary logistic regression. The final models were evaluated using the Hosmer–Lemeshow goodness-of-fit test. Associations between E-DII score and anatomical location (cardia or non-cardia) and E-DII score and histological subtype (diffuse or intestinal) were estimated by multinomial logistic regression. For both types of logistic regression, adjustments were made for multiple variables in different models. 

When considering cases and control I individuals, two models were tested, model 1—sex, age, marital status, education, self-reported ethnicity, family history of cancer in first-degree relatives, region studied, PPIs/H2RAs, antacids, aspirin, other NSAIDs, nutritional supplement, BMI (categories), tobacco smoking, alcohol consumption, diabetes, and sodium consumption (g/day); model 2—model 1 + peptic ulcer and *H. pylori* status. When analyzing cases and control II individuals, model 1 was selected. Quartile 1 (the most anti-inflammatory diet) was used as a reference for both analyses. 

The test for the linear trend was conducted by fitting the E-DII score as a continuous variable; the results from that model can be interpreted as the effect on GA outcomes for each 1-point-increment in E-DII score.

All tests were two-sided, and the significance level (α) was 5%. Analyses were performed using the software IBM SPSS^®^ Statistics version 25.0 (New York, NY, USA). 

## 3. Results

When compared with the two control groups, the cases group comprised mostly males (59.6%), individuals with a median age of 58 years, self-declared brown skin (50.6%), and with schooling ≤ 8 years (47.2%). About 61.6% of the cases had a family history of cancer in first-degree relatives, 43.1% showed intermediate/high tobacco smoking, and 37.4% reported intermediate/high alcohol consumption. In addition, 9.1% had diabetes, 8.3% peptic ulcer disease, and 22.6% had tested positive for *H. pylori* infection. In terms of drug use, 45.8% of cases consumed PPIs/H2RAs, 14.9% antacids, and 11.4% other NSAIDs; about 30% were overweight or obese. The cases had a median energy intake of 2193 kcal and sodium intake of 2.2 g/day, and values were higher than those in the control groups (*p* < 0.001). Most (77.7%) cases had GA located in the non-cardia region and 50.1% were classified as diffuse type. The overall E-DII score in this study ranged from −5.55 (maximum anti-inflammatory score) to +4.60 (maximum pro-inflammatory score). The cases group had higher median score (−0.45) when compared with both controls (−0.73 for control I and −0.83 for control II) (*p* < 0.001) (Table 1).

Cases in the highest quartile of the E-DII (Q4) were predominantly male (65.9%), younger (median age 56 years), declared themselves to be brown (49.6%), had diabetes (4.1%), and consumed aspirin (4.1%). These individuals had a higher sodium intake (median 6.2 g/day) when compared with lowest quartile (Q1) (*p* < 0.001). Control I participants in the Q4 were younger (median age 48 years), married (58.5%), had 9 to 12 years of schooling (44.7%), and a family history of cancer in first-degree relatives (58.5%). In addition, 40.4% had intermediate/high consumption of alcoholic beverages and 10.4% consumed nutritional supplements. These individuals had a higher energy (median 2328 kcal) and sodium (median of 1.8 g/day) intake when compared with Q1 (*p* < 0.001). Control II participants in Q4 were mostly male (58.8%), also younger (median 49 years), and 44.8% declared themselves to be brown. Moreover, 10.8% consumed PPIs/H2RAs, 3.1% antacids, 1.5% aspirin, and 8.8% nutritional supplements. Control II participants from Q4 also showed a higher sodium intake (median 4.8 g/day) when compared with Q1 (*p* < 0.001) (Table S1 from the Supplementary Material). 

Compared with both control groups, cases had a higher consumption of protein, SFAs and TFAs, cholesterol, vitamins B12 and D, selenium, and zinc; however, these individuals consumed less iron, omega-6, vitamins A, B1, B6, E, β-carotene, folic acid, fiber, magnesium, caffeine, and onion. The highest scores observed in the control I and control II quartiles indicated a more pro-inflammatory diet, with a lower intake of carbohydrates, iron, omega-3, vitamins A, B1, B2, B6, C, D, E, β-carotene, folic acid, fiber, magnesium, selenium, onion, and pepper. Q4 participants from both control groups consumed more pro-inflammatory compounds than anti-inflammatory ones compared to Q1 (Table 2).

When comparing cases and control I individuals, we observed that a more pro-inflammatory diet was 2.6 times as likely to be present in this instance than in the first quartile (OR_Q4vsQ1_: 2.60, 95% CI: 1.16–5.70, *p*-trend: 0.01). In Q3, there was a 2.1-fold increase in all GA when compared with Q1 (OR_Q3vsQ1_: 2.10, 95% CI: 1.06–3.98). An increased GA risk was observed with increasing E-DII score per 1-point increment (OR_E-DII_: 1.24, 95% CI: 1.05–1.46). Cases in the highest E-DII quartile were 3.93 times as likely to have GA in the non-cardia region (OR_Q4vsQ1_: 3.93, 95% CI: 1.59–9.70, *p*-trend: 0.01). This association was consistent when the non-cardia region was evaluated using the continuous E-DII variable per 1-point increment (OR_E-DII_: 1.28, 95% CI: 1.07–1.55). The pro-inflammatory diet was associated with increased GA of the diffuse subtype in Q3 and Q4 (OR_Q3vsQ1_: 2.36, 95% CI: 1.06–5.23; OR_Q4vsQ1_: 2.90, 95% CI: 1.06–7.82, *p*-trend: 0.01) and increasing E-DII score per 1-point increment (OR_E-DII_: 1.30, 95% CI: 1.06–1.60) (Table 3).

The risk of having GA was 2.7 times as likely in participants eating the most pro-inflammatory diets compared to those consuming the most more anti-inflammatory diets (OR_Q4vsQ1_: 2.70, 95% CI: 1.60–4.54, *p*-trend: <0.001). In Q3, the risk of GA was 2.27-fold increase that of Q1 (OR_Q3vsQ1_: 2.27, 95% CI: 1.44–3.58). An increased GA risk was observed with increasing E-DII score per 1-point increment (OR_E-DII_: 1.27, 95% CI: 1.13–1.43). There was a >3-fold risk of GA in the cardia region associated with the most pro-inflammatory diet (OR_Q4vsQ1_: 3.31, 95% CI: 1.32–8.24, *p*-trend: 0.007) and increasing E-DII score per 1-point increment (OR_E-DII_: 1.31, 95% CI: 1.08–1.60). There also was an increase in GA in the non-cardia region in Q3 and Q4 compared with Q1 (OR_Q3vsQ1_: 2.43, 95% CI: 1.44–4.09; OR_Q4vsQ1_: 2.97, 95% CI: 1.64–5.39, *p*-trend: <0.001; OR_E-DII_: 1.30, 95% CI: 1.14–1.50). Those individuals in the highest E-DII quartile had a higher risk of GA of the diffuse and intestinal subtypes (OR_Q4vsQ1_: 2.48, 95% CI: 1.23–5.00, *p*-trend: 0.003 for diffuse; OR_Q4vsQ1_: 2.82, 95% CI: 1.38–5.74, *p*-trend: 0.002 for intestinal). Although there was an increased risk for both subtypes in E-DII quartile 3, it was highest for the diffuse subtype (OR_Q3vsQ1_: 2.80, 95% CI: 1.54–5.10). This association was consistent when diffuse and intestinal GA subtypes were evaluated using the continuous E-DII variable per 1-point increment (OR_E-DII_: 1.26, 95% CI: 1.08–1.48, and OR_E-DII_: 1.30, 95% CI: 1.10–1.51; respectively) (Table 4).

## 4. Discussion

There was a positive association between E-DII score and GA in the Brazilian population. A pro-inflammatory diet was associated with an increased risk of cardia and non-cardia GA of both intestinal and diffuse subtypes. 

Our findings are consistent with those found in populations from Italy (OR: 2.35; 95% CI: 1.32–4.20) [34], South Korea (OR: 1.63; 95% CI: 1.15–2.29) [11], and Iran (OR: 3.39; 95% CI: 1.59–7.22) [35]. These results are also consistent with those from a prospective cohort conducted in Sweden, in which 163 cases of GC (92 cases in men and 71 in women) were diagnosed, and a low-DII diet was associated with a lower risk of GC just in men (HR: 0.73; 95% CI: 0.53–0.99) [36]. In yet another cohort conducted in the US, no association was observed between E-DII score and either cardia or non-cardia GA [37]. 

In the subgroup analyses based on histological subtype in cases versus control I individuals, we observed an increased risk of diffuse GA. A minority (10%) of diffuse GA is associated with genetic alterations and family history, while 80 to 90% of such cases are considered “sporadic” [5,38]. The association between *H. pylori* infection and the sporadic diffuse type (without association with atrophic gastritis and intestinal metaplasia) remains an enigma [39]. It has been recently shown that the diffuse subtype shares co-factors usually associated with the non-diffuse subtype, such as smoking, high salt intake, and low fruit and vegetable intake. Notably, some high-risk countries for GA in East Asia and Central America report that approximately 50% of GA belong to the diffuse subtype. In the US, the occurrence of this subtype was higher in young adults between 2000 and 2019 [5], which corroborates our own findings demonstrating a higher prevalence of the diffuse subtype in the Brazilian population. The risk of diffuse GA in this study was found to be associated with a more pro-inflammatory diet among adults < 50 years of age.

Countries with high incidence of GA, such as Japan and South Korea, have national prevention programs, such as endoscopic screening, for early detection of this type of cancer in the asymptomatic population, which has reduced mortality caused by GA [4,40]. Brazil lacks an organized GA detection program. Thus, although the incidence rates in Brazil are one quarter of those recorded in Japan and South Korea, mortality from GA is high, which demonstrates the need for preventive activities and early diagnosis in an organized manner. 

Based on histological subtype, when comparing cases with the control II group we observed an increased risk of intestinal GA, for which the main risk factor is *H. pylori* infection, whose prevalence in Latin America and the Caribbean is one of the largest in the world—69.26% in adults [4,41]. Gastric carcinogenesis caused by *H. pylori* infection was described by Correa and Piazuelo [42]. It is now common knowledge that diet can influence the inflammatory process or inhibit the inflammatory cascade. Thus, a synergy between infection and environmental carcinogens (nitrosamines, heterocyclic amines, nitrites, dietary salt, alcohol) may occur, not to mention complex interactions with antioxidants, resulting in decreased protective effects and promotion of carcinogenesis. Daily consumption of fruits and vegetables, which are sources of antioxidant/anti-inflammatory nutrients (vitamin E, ascorbic acid, and β-carotene), as well as allium vegetables that contain flavonoids and organosulfur compounds, promote gastric cyto-protection, and can reverse premalignant lesions [6,43,44,45]. Additionally, regular use of nutritional supplementation was associated with reducing risk of CG and non-cardia CG, regardless of lifestyle [46,47,48]. However, use of high doses of anti-inflammatory nutrients, such as β-carotene, vitamin C, and vitamin E, for cancer prevention purposes in generally healthy populations has not shown beneficial effects on incidence and mortality, regardless of cancer type [49]. In this study, we observed a more pro-inflammatory dietary pattern in the Brazilian population, with lower consumption of the anti-inflammatory nutrients. 

A case–control study carried out in Korea with 1164 adults (388 cases and 776 controls) was the only one so far to associate a pro-inflammatory diet—represented by the highest tertile of DII in relation to the lowest tertile of the histological GA subtypes—with a higher risk of the intestinal subtype in men (OR = 2.03, 95% CI: 1.09–3.77) and the intestinal and diffuse subtypes in women (OR = 4.87, 95% CI: 1.47–16.07 for intestinal, OR = 2.93, 95% CI: 1.47–5.84 for diffuse) [11]. Our study is a pioneering contribution to the literature on the risk of GA stratified by histological subtypes, presenting a comparison between cases and two controls groups. Our findings indicate that a pro-inflammatory diet is associated with a higher risk of diffuse GA in the endoscopic control, which may be related to early detection of intestinal GA by endoscopy. 

The central-western region of Brazil is internationally known for its agrobusiness sector, with the production of soy, corn, sugar cane, and cattle. This region is responsible for 41% of the Brazilian agricultural production, although fruit production is incipient [50,51]. However, organic agriculture in this region is still limited, and it is known that activities involving the application of pesticides can contribute to carcinogenesis [52]. In the 2017–2018 biennium, the inclusion of minimally processed foods in the Brazilian population’s diet was higher in the northern region and lower in the southeastern and central-western regions. The consumption of ultra-processed foods was higher in the southeastern and central-western regions and lower in the northern region of Brazil. The diet of the Brazilian population is still predominantly composed of fresh, minimally processed foods and processed culinary ingredients. However, there has been an increase in the consumption of ultra-processed foods [53], mainly in adults [54]. The consumption of processed and ultra-processed foods was associated with GA in individuals from Belém and São Paulo [30]. In addition, early onset GC of diffuse subtype was identified in younger adults [4]. Our results corroborate these findings, as we observed a higher consumption of a pro-inflammatory diet and a decrease in the consumption of an anti-inflammatory diet, especially among adults. However, a lower E-DII score was observed in individuals aged over 50 years, which may be related to the presence of comorbidities that motivate changes in diet. Moreover, the association of ultra-processed foods with low-grade inflammation is only partially explained by the high pro-inflammatory potential of these foods [55].

In this study, approximately 50% of hospital controls were overweight (overweight and obese), which contributes to the increased risk of cardia GA. Metabolically active visceral adipose tissues produce inflammatory mediators and cytokines (e.g., TNF-α and leptin), inhibit adiponectin secretion, and facilitate the development of insulin resistance and subsequent hyperinsulinemia, promoting carcinogenesis partly by stimulating an increase in insulin-like growth factor 1 (IGF-1) expression [43]. It has been reported that the level of adiponectin is lower in patients with cardia GA than in control individuals [56]. With the consumption of a predominantly pro-inflammatory diet, the release of inflammatory mediators can be potentiated and contribute to carcinogenesis. 

Sodium intake by cases and hospital controls who consumed a more pro-inflammatory diet was more than twice the maximum recommended by the WHO (2 g/day). In 2021, the Pan American Health Organization launched new targets to reduce average salt/sodium consumption by 30% in the population of the Americas by 2025 [57,58]. In addition to the risk factors cited here and despite the efforts to reduce sodium consumption, the Brazilian population in this study displayed high sodium intake, which may contribute to gastric carcinogenesis.

The strengths of this study are (1) its relatively large sample size in which both diet and GA were analyzed; (2) it is the first Brazilian multicenter case–control study that uses the E-DII tool; and (3) it is the first multicenter study that includes individuals from three capitals located in three different regions of Brazil. Despite its strengths, this study has limitations, such as (1) recall and selection biases that are frequent in case–control studies; (2) the reverse causation bias (in case–control study), as a diet may be affected by the diagnosis of GA; and (3) another limitation to be considered is the use of the NDSR^®^ software for dietary nutritional analysis of the participants. As it is a US food database, it may not reliably reflect the nutritional composition of Brazilian foods; however, it is considered a robust and complete nutrient database, widely used in epidemiological studies both in Brazil and in other countries. Because there is no Brazilian nutritional composition table with these characteristics, its use was adopted with the inclusion of typically national foods. 

## 5. Conclusions

In conclusion, a pro-inflammatory diet, estimated by higher E-DII scores, was associated with an increased risk of GA in both the cardia and non-cardia anatomic regions and both intestinal and diffuse histological subtypes in this Brazilian population. However, E-DII needs improvement for GA with the inclusion of nutrients, such as sodium, given the importance of the nutrient in carcinogenesis. Additional studies on inflammatory diet and GA in this population are needed to better understand the relationship between anti-inflammatory components of the Brazilian diet and GA.

## Figures and Tables

**Table 1:** Sociodemographic, clinical, and nutritional characteristics of cases and controls, GE4GAC-Brazil (2016–2022).

Variables	GE4GAC-Brazil (*n* = 1645)			*p*-Value ^1^
	Cases (*n* = 492)	Control I (*n* = 377)	Control II (*n* = 776)	
	Median (P_25_, P_75_) or *n* (%)			
*Sex*				0.002
*Female*	199 (40.4)	182 (48.3)	398 (51.3)	
*Male*	293 (59.6)	195 (51.7)	378 (48.7)	
*Age (years)*	58 (49, 65) ^a^	57 (44, 64) ^b^	55 (44, 64) ^b^	0.001
*Self-reported ethnicity*				
*White*	181 (36.8)	180 (47.7)	287 (37.0)	<0.001
*Brown*	249 (50.6)	134 (35.5)	320 (41.3)	
*Black*	44 (8.9)	43 (11.4)	110 (14.2)	
*Others*	18 (3.7)	20 (5.3)	58 (7.5)	
*Schooling (years)* †				<0.001
*≤8*	232 (47.2)	96 (25.5)	172 (22.2)	
*9–12*	159 (32.3)	163 (43.2)	412 (53.1)	
*≥13*	101 (20.5)	118 (31.3)	192 (24.7)	
*Marital status*				0.13
*Married*	353 (71.7)	247 (65.5)	523 (67.4)	
*Single*	71 (14.4)	66 (17.5)	147 (18.9)	
*Others*	68 (13.8)	64 (17.0)	106 (13.7)	
*Family history of cancer in first-degree relatives*				<0.001
*No*	188 (38.4)	159 (42.3)	391 (50.5)	
*Yes*	302 (61.6)	217 (57.7)	383 (49.5)	
*Tobacco smoking* §				<0.001
*No*	198 (40.5)	227 (60.5)	495 (64.1)	
*Low*	80 (16.4)	67 (17.9)	106 (13.7)	
*Intermediate/High*	211 (43.1)	81 (21.6)	171 (22.2)	
*Alcohol consumption* ¥				<0.001
*No*	230 (47.2)	180 (48.5)	508 (66.7)	
*Low*	75 (15.4)	55 (14.8)	64 (8.4)	
*Intermediate/High*	182 (37.4)	136 (36.7)	190 (24.9)	
*Diabetes*				0.003
*No*	447 (90.9)	327 (86.7)	721 (92.9)	
*Yes*	45 (9.1)	50 (13.3)	55 (7.1)	
*Peptic ulcer*				0.04
*No*	451 (91.7)	359 (95.2)	-	
*Yes*	41 (8.3)	18 (4.3)	-	
*H. pylori status* *				0.04
*Negative*	230 (77.4)	226 (70.0)	-	
*Positive*	67 (22.6)	97 (30.0)	-	
*PPIs/H2RAs*				<0.001
*No*	265 (54.2)	215 (57.2)	681 (88.0)	
*Yes*	224 (45.8)	161 (42.8)	93 (12.0)	
*Antacids*				<0.001
*No*	418 (85.1)	329 (87.5)	755 (97.4)	
*Yes*	73 (14.9)	47 (12.5)	20 (2.6)	
*Aspirin*				0.33
*No*	462 (94.1)	351 (93.4)	739 (95.4)	
*Yes*	29 (5.9)	25 (6.6)	36 (4.6)	
*Other NSAIDs*				<0.001
*No*	435 (88.6)	331 (88.0)	736 (95.0)	
*Yes*	56 (11.4)	45 (12.0)	39 (5.0)	
*BMI (categories)*				<0.001
*Normal weight*	208 (42.3)	148 (39.4)	322 (41.5)	
*Underweight/Malnutrition*	138 (28.0)	24 (6.4)	88 (11.4)	
*Overweight*	78 (15.9)	118 (31.4)	222 (28.6)	
*Obesity*	68 (13.8)	86 (22.9)	143 (18.5)	
*Energy intake (kcal/day)*	2193 (1664, 2824) ^a^	2087 (1503, 2653) ^a^	1792 (1352, 2391) ^b^	<0.001
*Sodium intake (g/day)*	2.2 (1.7, 5.5) ^a^	1.7 (1.5, 2.0) ^c^	1.8 (1.5, 2.5) ^b^	<0.001
*Nutritional supplement*				0.07
*No*	408 (83.1)	308 (81.7)	671 (86.5)	
*Yes*	83 (16.9)	69 (18.3)	105 (13.5)	
*Anatomical location*				-
*Cardia*	101 (22.3)	-	-	
*Non-cardia*	352 (77.7)	-	-	
*Histological subtype*				-
*Diffuse*	203 (50.1)	-	-	
*Intestinal*	174 (43.0)	-	-	
*Mixed*	28 (6.9)	-	-	
*E-DII score*	−0.45 (−1.46, 0.53) ^b^	−0.73 (−2.00, 0.56) ^a^	−0.83 (−2.03, 0.25) ^a^	<0.001

Numbers may differ because of missing values. Control I individuals (endoscopy); Control II individuals (hospital). PPIs/H2RAs: proton pump inhibitors/H2-receptor antagonists; NSAIDs: non-steroidal anti-inflammatory drugs; BMI: body mass index; E-DII: energy-adjusted dietary inflammatory index. † ≤8 years = Illiterate to elementary school, 9 to 12 years = High school and ≥ 13 years = Higher education. § Low smoking: ≤ 10 packs/year and Intermediate/High smoking: >10 packs/year. ¥ Low: ≤12 g/day and Moderate/High: >12 g/day alcohol. * 195 cases and 54 control I individuals were not tested. ^1^ Pearson χ^2^ test for categorical variables and Kruskal–Wallis test and Dunn–Bonferroni post hoc test for continuous variables. Different letters on the same line mean statistical difference between groups. Significance *p*-value < 0.05.

**Table 2:** Dietary intake of the participants, GE4GAC-Brazil (2016–2022).

Components	Cases (*n* = 492)	Control I (*n* = 377)	Control II (*n* = 776)	*p*-Value ^1^	E-DII Quartiles §									
					Control I					Control II				
					Q1 *n* = 94 (−5.55, −2.01)	Q2 *n* = 94 (−2.00, −0.74)	Q3 *n* = 95 (−0.73, 0.56)	Q4 *n* = 94 (>0.56)	*p*-Value ^1^ ‡	Q1 *n* = 194 (−5.09, −2.04)	Q2 *n* = 194 (−2.03, −0.83)	Q3 *n* = 194 (−0.82, 0.25)	Q4 *n* = 194 (>0.25)	*p*-Value ^1^ ‡
	Median (P_25_, P_75_)				Median (P_25_, P_75_)					Median (P_25_, P_75_)				
***Pro-inflammatory***														
*Carbohydrates (g/day)*	119.7 (104.8, 133.4)	122.1 (107.8, 136.5)	121.5 (106.5, 136.4)	0.23	131.8 (118.4, 147.3) ^bc^	122.8, 113.6, 135.7) ^e^	118.2 (105.8, 134.0)	112.7 (97.0, 126.1)	<0.001	130.4 (115.6, 147.2) ^bc^	126.0 (112.7, 141.1) ^de^	115.5 (101.7, 130.0)	111.7 (96.0, 125.4)	<0.001
*Proteins (g/day)*	43.7 (38.9, 49.5) ^a^	41.5 (37.5, 47.1) ^b^	43.0 (37.5, 48.7) ^a^	<0.001	40.7 (36.3, 45.5)	42.0 (36.2, 46.8)	40.2 (36.7, 46.6)	43.0 (36.4, 49.5)	0.45	42.6 (36.7, 43.4) ^bc^	41.7 (36.1, 46.9) ^de^	44.0 (39.1, 51.1)	44.1 (38.1, 51.1)	<0.001
*Total fats (g/day)*	39.9 (35.1, 44.4)	40.4 (35.4, 44.8)	39.8 (34.7, 44.5)	0.54	36.9 (32.6, 43.5) ^c^	39.4 (35.3, 43.2) ^e^	41.4 (36.7, 45.0)	42.7 (39.3, 46.8)	<0.001	38.5 (33.4, 43.3) ^c^	38.6 (33.6, 43.0) ^e^	40.6 (35.3, 44.8)	41.5 (38.0, 46.3)	<0.001
*SFAs (g/day)*	12.7 (11.0, 14.2) ^a^	12.2 (10.3, 14.3) ^ab^	12.2 (10.5, 14.2) ^bc^	0.03	10.8 (9.0, 12.6) ^bc^	11.6 (9.9, 13.4) ^e^	12.6 (10.8, 14.3) ^f^	14.3 (12.5, 15.3)	<0.001	10.9 (9.7, 12.7) ^bc^	11.7 (10.1, 13.5) ^de^	12.7 (10.9, 14.4) ^f^	13.8 (11.7, 15.3)	<0.001
*TFAs (g/day)*	0.9 (0.7, 1.1) ^ab^	0.9 (0.7, 1.2) ^ab^	0.8 (0.6, 1.1) ^c^	0.001	0.6 (0.5, 0.9) ^bc^	0.9 (0.6, 1.1) ^e^	1.0 (0.8, 1.2) ^f^	1.2 (0.9, 1.4)	<0.001	0.7 (0.5, 0.9) ^bc^	0.8 (0.6, 0.9) ^de^	0.9 (0.7, 1.1)	1.0 (0.8, 1.2)	<0.001
*Cholesterol (mg/day)*	153.2 (119.6, 196.8) ^a^	131.1 (102.0, 166.0) ^c^	144.5 (110.2, 187.0) ^b^	<0.001	115.4 (96.0, 163.5) ^c^	128.2 (96.4, 155.5)	135.6 (108.4, 161.0)	143.6 (118.0, 178.3)	0.01	128.6 (99.0, 175.0) ^bc^	130.6 (102.2, 170.7) ^de^	152.6 (115.6, 198.0)	159.2 (125.0, 199.7)	<0.001
*Iron (mg/day)*	6.2 (5.6, 7.0) ^b^	6.5 (5.7, 7.4) ^a^	6.4 (5.6, 7.2) ^ab^	0.03	6.5 (5.8, 7.3)	6.7 (5.8, 7.5) ^e^	6.9 (6.0, 7.6) ^f^	6.1 (5.6, 6.7)	0.001	7.0 (5.9, 7.8) ^abc^	6.4 (5.6, 7.3) ^e^	6.3 (5.6, 6.9)	6.0 (5.3, 6.7)	<0.001
*Vitamin B12 (mg/day)*	1.9 (1.5, 2.3) ^a^	1.7 (1.3, 2.0) ^c^	1.8 (1.4, 2.3) ^b^	<0.001	1.5 (1.2, 1.9) ^c^	1.7 (1.2, 1.9) ^e^	1.7 (1.4, 2.1)	1.8 (1.5, 2.4)	<0.001	1.6 (1.2, 2.0) ^bc^	1.7 (1.3, 2.1) ^de^	1.8 (1.5, 2.3)	2.0 (1.6, 2.5)	<0.001
***Anti-inflammatory***														
*Omega-3 (g/day)*	1.2 (1.1, 1.5)	1.2 (1.0, 1.5)	1.3 (1.1, 1.5)	0.14	1.4 (1.1, 1.6) ^bc^	1.2 (1.1, 1.4)	1.2 (1.0, 1.4) ^f^	1.1 (0.9, 1.3)	<0.001	1.3 (1.1, 1.6) ^bc^	1.2 (1.1, 1.5)	1.2 (1.0, 1.4)	1.2 (1.0, 1.4)	0.001
*Omega-6 (g/day)*	7.8 (6.6, 9.1) ^b^	8.1 (7.0, 9.5) ^a^	8.0 (6.8, 9.5) ^b^	0.01	8.2 (7.0, 9.5)	8.2 (7.0, 9.3)	8.1 (7.1, 9.4)	8.0 (6.4, 9.6)	0.76	8.4 (6.9, 9.9)	8.0 (6.8, 9.6)	8.0 (6.7, 9.3)	7.8 (6.5, 9.1)	0.076
*MUFAs (g/day)*	13.4 (11.6, 15.1)	13.7 (11.6, 15.8)	13.5 (11.4, 15.4)	0.21	12.7 (10.7, 16.2) ^c^	13.3 (11.4, 15.0) ^e^	13.7 (11.7, 15.4)	14.6 (13.1, 16.4)	0.003	13.4 (11.2, 15.3)	13.1 (11.0, 14.8) ^e^	13.6 (11.5, 15.4)	14.3 (12.2, 15.8)	0.008
*Vitamin A (RE/day)*	258.1 (195.7, 348.5) ^c^	322.4 (234.1, 432.1) ^a^	280.1 (209.8, 387.0) ^b^	<0.001	374.4 (272.3, 577.3) ^c^	336.3 (231.1, 502.7) ^e^	327.2 (258.3, 418.0) ^f^	245.1 (190.6, 348.6)	<0.001	384.7 (294.9, 560.7) ^abc^	284.0 (211.7, 373.1) ^de^	246.0 (186.0, 340.2)	238.2 (180.1, 318.6)	<0.001
*β* *-carotene (µg/day)*	982.7 (610.2, 1476.3) ^b^	1257.6 (809.7, 1833.0) ^a^	1144.3 (700.5, 1786.6) ^a^	<0.001	2005.6 (1581, 2820.0) ^abc^	1437.2 (1011.3, 1931.0) ^de^	1082.5 (817.7, 1454.1) ^f^	714.8 (489.0, 1038.6)	<0.001	2205.6 (1578.3, 3041.6) ^abc^	1245.8 (862.7, 1646.5) ^de^	922.0 (567.6, 1251.1)	745.4 (544.3, 1093.6)	<0.001
*Vitamin B1 (mg/day)*	0.7 (0.6, 0.8) ^b^	0.8 (0.7, 0.9) ^a^	0.7 (0.6, 0.8) ^ab^	0.003	0.8 (0.7, 0.9) ^bc^	0.8 (0.7, 0.9) ^e^	0.8 (0.7, 0.8) ^f^	0.7 (0.6, 0.8)	<0.001	0.8 (0.7, 0.9) ^abc^	0.8 (0.7, 0.9) ^de^	0.7 (0.6, 0.8)	0.7 (0.6, 0.8)	<0.001
*Vitamin B2 (mg/day)*	0.7 (0.6, 0.8)	0.7 (0.6, 0.8)	0.7 (0.6, 0.8)	0.38	0.8 (0.7, 0.9) ^bc^	0.7 (0.6, 0.8)	0.7 (0.6, 0.8)	0.7 (0.6, 0.8)	<0.001	0.8 (0.7, 0.9) ^abc^	0.7 (0.6, 0.8)	0.7 (0.6, 0.8)	0.7 (0.6, 0.8)	<0.001
*Niacin (mg/day)*	10.0 (8.6, 11.4)	9.8 (8.3, 11.2)	9.8 (8.4, 11.4)	0.13	9.6 (8.2, 11.2)	9.8 (8.3, 11.4)	9.3 (8.3, 11.1)	10.0 (8.6, 11.7)	0.39	9.5 (7.9, 10.6) ^bc^	9.4 (7.9, 10.6) ^de^	10.4 (9.1, 12.1)	10.3 (8.5, 11.9)	<0.001
*Vitamin B6 (mg/day)*	1.0 (0.9, 1.2) ^a^	1.0 (0.8, 1.2) ^b^	1.0 (0.9, 1.2) ^a^	0.002	1.1 (1.0, 1.3) ^abc^	1.0 (0.9, 1.1)	0.9 (0.8, 1.1)	0.9 (0.7, 1.0)	<0.001	1.1 (1.0, 1.2) ^abc^	1.0 (0.9, 1.2)	1.0 (0.9, 1.2)	1.0 (0.9, 1.1)	<0.001
*PUFAs (g/day)*	9.3 (8.2, 11.0)	9.8 (8.3, 11.4)	9.7 (8.2, 11.3)	0.06	9.8 (8.3, 11.5)	9.8 (8.4, 11.2)	9.8 (8.4, 11.1)	9.6 (7.9, 11.2)	0.67	10.2 (8.4, 11.9)	9.7 (8.3, 11.5)	9.6 (8.1, 11.0)	9.5 (8.1, 10.8)	0.090
*Folic acid (µg/day)*	214.5 (184.8, 256.3) ^c^	235.7 (202.0, 274.5) ^a^	227.0 (190.5, 266.2) ^b^	<0.001	267.5 (224.8, 306.7) ^bc^	247.5 (219.0, 285.7) ^e^	236.7 (209.7, 267.3) ^f^	196.0 (172.7, 223.8)	<0.001	256.2 (229.1, 302.5) ^abc^	239.8 (208.6, 279.2) ^de^	216.3 (186.1, 249.0) ^f^	193.8 (169.8, 224.8)	<0.001
*Vitamin C (mg/day)*	57.3 (33.6, 83.5)	54.8 (34.0, 94.4)	59.0 (36.7, 93.1)	0.11	111.1 (87.8, 150.0) ^abc^	69.0 (51.3, 95.7) ^de^	45.6 (30.7. 58.5) ^f^	32.1 (23.2, 42.2)	<0.001	106.5 (74.4, 145.3) ^abc^	70.7 (49.6, 99.9) ^de^	46.4 (31.5, 62.8)	38.2 (25.7, 53.4)	<0.001
*Vitamin D (µg/day)*	3.0 (2.0, 4.3) ^a^	2.0 (1.4, 2.9) ^c^	2.6 (1.7, 3.8) ^b^	<0.001	2.6 (1.8, 3.3) ^bc^	2.1 (1.4, 3.0)	1.8 (1.3, 2.7)	1.8 (1.4, 2.3)	<0.001	2.8 (1.8, 3.8)	2.4 (1.6, 3.4)	2.6 (1.5, 3.7)	2.6 (1.8, 4.2)	0.092
*Vitamin E (mg/day)*	3.2 (2.8, 3.7) ^b^	3.6 (3.1, 4.3) ^a^	3.5 (2.9, 4.2) ^a^	<0.001	4.6 (4.0, 5.2) ^abc^	3.6 (3.3, 4.3) ^de^	3.3 (3.0, 3.7) ^f^	3.0 (2.6, 3.5)	<0.001	4.3 (3.9, 5.0) ^abc^	3.6 (3.1, 4.3) ^de^	3.1 (2.8, 3.6)	3.0 (2.6, 3.4)	<0.001
*Fibers (g/day)*	10.5 (8.3, 13.3) ^b^	11.8 (9.4, 14.8) ^a^	11.4 (8.6, 14.5) ^a^	<0.001	16.0 (14.2, 19.1) ^abc^	13.1 (11.5, 15.2) ^de^	10.8 (9.5, 12.7) ^f^	8.3 (7.1, 9.7)	<0.001	16.1 (13.3, 20.3) ^abc^	12.8 (10.9, 15.0) ^de^	10.1 (8.2, 11.6) ^f^	8.2 (7.0, 10.0)	<0.001
*Magnesium (g/day)*	133.7 (119.8, 156.6) c	139.5 (121.0, 163.3) abc	141.0 (122.4, 165.5) ab	0.01	175.8 (157.0, 200.2) ^abc^	146.4 (133.2, 163.3) ^de^	131.5 (122.8, 143.3) ^f^	114.4 (102.7, 123.3)	<0.001	175.7 (152.9, 218.4) ^abc^	152.8 (133.1, 167.2) ^de^	129.8 (118.1, 141.8)	121.0 (109.6, 134.2)	<0.001
*Selenium (g/day)*	62.1 (54.2, 75.0) ^a^	58.8 (51.4, 71.2) ^c^	61.8 (52.8, 75.2) ^ab^	0.003	59.0 (50.8, 75.4)	58.4 (51.5, 68.0)	58.3 (52.4, 72.4)	60.3 (51.3, 70.2)	0.79	66.2 (54.1, 78.9) ^c^	59.8 (50.5, 73.0)	62.6 (53.7, 72.7)	61.1 (53.1, 71.1)	0.018
*Zinc (mg/day)*	6.5 (5.7, 7.4) ^a^	6.3 (5.4, 7.2) ^ab^	6.3 (5.5, 7.2) ^b^	0.03	6.0 (5.3, 6.9)	6.5 (5.4, 7.3)	6.5 (5.8, 7.2)	6.4 (5.7, 7.5)	0.13	6.2 (5.3, 6.8) ^bc^	6.1 (5.3, 7.3) ^e^	6.5 (5.8, 7.4)	6.6 (5.7, 7.4)	0.001
*Caffeine (mg/day)*	0.6 (0.1, 2.7) ^b^	2.0 (0.4, 23.0) ^a^	0.6 (0.9, 3.3) ^b^	<0.001	0.4 (0.0, 1.6) ^abc^	1.5 (0.2, 22.8) ^e^	7.8 (0.8, 28.0)	14.5 (1.4, 31.0)	<0.001	0.5 (0.5, 1.7)	0.7 (0.2, 2.9)	0.6 (0.1, 4.9)	0.8 (0.0, 5.3)	0.470
*Onion (g/day)*	2.5 (0.0, 6.1) ^c^	3.9 (1.6, 8.4) ^a^	3.5 (0.6, 6.5) ^b^	<0.001	5.7 (2.1, 12.0) ^c^	4.5 (1.2, 9.3)	4.0 (1.8, 8.4)	2.9 (1.2, 5.1)	0.001	5.1 (2.1, 9.4) ^bc^	4.0 (1.8, 6.4) ^de^	2.7 (0.0, 5.6)	2.2 (0.0, 5.0)	<0.001
*Pepper (g/day)*	0.0 (0.0, 0.8)	0.0 (0.0, 0.4)	0.0 (0.0, 0.7)	0.21	0.0 (0.0, 0.0)	0.0 (0.0, 0.5)	0.0 (0.0, 0.8)	0.0 (0.0, 0.5)	0.15	0.0 (0.0, 0.6)	0.0 (0.0, 0.2) ^d^	0.0 (0.0, 0.8)	0.0 (0.0, 0.9)	0.016

Control I individuals (endoscopy); Control II individuals (hospital). § Based on control I and control II scores. SFAs: saturated fatty acids; TFAs: trans fatty acids; MUFAs: monounsaturated fatty acids; PUFAs: polyunsaturated fatty acids. ^1^ Kruskal–Wallis test and Dunn–Bonferroni post hoc test. Significance *p*-value < 0.05. ‡ Difference between quartiles: ^a^ Quartiles 1 × 10^2^; ^b^ Quartiles 1 × 10^3^; ^c^ Quartiles 1 × 10^4^; ^d^ Quartiles 2 × 10^3^; ^e^ Quartiles 2 × 10^4^; ^f^ Quartiles 3 × 10^4^.

**Table 3:** Odds ratios between energy-adjusted Dietary Inflammatory Index (E-DII) and gastric adenocarcinoma by anatomical location and histological subtype in cases versus control I individuals, GE4GAC-Brazil (2016–2022).

	E−DII Quartiles					*p*−Trend ‡
	Q1 (−5.55, −2.01)	Q2 (−2.00, −0.74)	Q3 (−0.73, 0.56)	Q4 (>0.56)	Per 1-Point Increment in the E-DII Score	
	OR	OR (95% CI)	OR (95% CI)	OR (95% CI)		
***All*** **^1^**						
* Cases/Controls*	68/94	134/94	169/95	121/94	492/377	
* Crude*	1.00	1.97 (1.31–2.96)	2.46 (1.65–3.67)	1.78 (1.18–2.68)	1.15 (1.05–1.26)	0.003
* Model 1*	1.00	1.62 (0.97–2.70)	1.94 (1.14–3.30)	1.70 (0.88–3.27)	1.17 (1.02–1.35)	0.02
* Model 2* *^a^*	1.00	1.51 (0.81–2.81)	2.10 (1.06–3.98)	2.60 (1.16–5.70)	1.24 (1.05–1.46)	0.01
***Anatomical location*** **^2^**						
***Cardia***						
* Cases/Controls*	13/94	32/94	30/95	26/94	101/377	
* Crude*	1.00	2.46 (1.22–4.98)	2.28 (1.12–4.65)	2.00 (0.96–4.13)	1.19 (1.03–1.38)	0.02
* Model 1*	1.00	1.90 (0.85–4.20)	2.02 (0.87–4.64)	1.81 (0.69–4.73)	1.26 (1.03–1.54)	0.03
* Model 2*	1.00	1.22 (0.50–2.97)	1.49 (0.58–3.83)	1.49 (0.49–4.53)	1.19 (0.95–1.50)	0.13
***Non-cardia***						
* Cases/Controls*	49/94	93/94	126/95	84/94	352/377	
* Crude*	1.00	1.90 (1.21–2.97)	2.54 (1.65–3.94)	1.71 (1.09–2.70)	1.13 (1.03–1.25)	0.01
* Model 1*	1.00	1.60 (0.89–2.88)	2.28 (1.24–4.19)	1.82 (0.86–3.88)	1.19 (1.02–1.40)	0.03
* Model 2*	1.00	1.75 (0.86–3.60)	2.54 (1.19–5.41)	3.93 (1.59–9.70)	1.28 (1.07–1.55)	0.01
***Histological subtype*** **^2^**						
***Diffuse***						
* Cases/Controls*	29/94	55/94	76/95	43/94	203/377	
* Crude*	1.00	1.90 (1.11–3.23)	2.59 (1.55–4.34)	1.48 (0.86–2.57)	1.12 (0.99–1.25)	0.06
* Model 1*	1.00	1.68 (0.87–3.17)	2.43 (1.26–4.68)	2.30 (1.00–5.23)	1.23 (1.07–1.51)	0.01
* Model 2*	1.00	1.45 (0.67–3.13)	2.36 (1.06–5.23)	2.90 (1.06–7.82)	1.30 (1.06–1.60)	0.01
***Intestinal***						
* Cases/Controls*	25/94	48/94	62/95	39/94	174/377	
* Crude*	1.00	1.92 (1.10–3.37)	2.45 (1.42–4.23)	1.56 (0.87–2.78)	1.13 (0.99–1.23)	0.06
* Model 1*	1.00	1.93 (0.98–3.84)	2.34 (1.15–4.77)	1.53 (0.63–3.70)	1.20 (1.00–1.44)	0.05
* Model 2*	1.00	1.75 (0.75–4.10)	2.16 (0.88–5.30)	2.58 (0.89–7.44)	1.26 (1.00–1.57)	0.04

^1^ Binary logistic regression. ^2^ Multinomial logistic regression. The mixed histological subtype was not considered because the number of cases was <30. Reference: Quartile 1 (maximum anti-inflammatory diet). OR: Odds ratio. 95% CI: 95% confidence interval. Model 1: adjusted for sex, age (years), marital status, schooling, self-reported ethnicity, family history of cancer in first-degree relatives, study region, PPIs/H2RAs, antacids, NSAIDs, aspirin, nutritional supplement, BMI (categories), tobacco smoking, alcohol beverage consumption, diabetes, sodium intake (g/day). Model 2: Model 1 + peptic ulcer, *H.pylori* status. ^a^ Hosmer–Lemeshow test = 13.35, *p* = 0.10 (E-DII quartiles) and 12.08, *p* = 0.15 (E-DII continuous). ‡ *p*-value for trend derived from models using the E-DII continuous variable.

**Table 4:** Odds ratios between Energy-adjusted Dietary Inflammatory Index (E-DII) and gastric adenocarcinoma by anatomical location and histological subtype in cases versus controls II, GE4GAC-Brazil (2016–2022).

	E-DII Quartiles				Per 1-Point Increment in theE-DII Score	
	Q1 (−5.09, −2.04)	Q2 (−2.03, −0.83)	Q3 (−0.82, 0.25)	Q4 (>0.25)		*p*-Trend ‡
	OR	OR (95% CI)	OR (95% CI)	OR (95% CI)		
***All ^1^***						
* Cases/Controls*	65/194	119/194	156/194	152/195	492/777	
* Crude*	1.00	1.83 (1.28–2.65)	2.41 (1.69–3.44)	2.35 (1.65–3.36)	1.20 (1.11–1.29)	<0.001
* Model 1 ^a^*	1.00	1.42 (0.91–2.22)	2.27 (1.44–3.58)	2.70 (1.60–4.54)	1.27 (1.13–1.43)	<0.001
***Anatomical location ^2^***						
***Cardia***						
* Cases/Controls*	11/194	30/194	24/194	36/195	101/777	
* Crude*	1.00	2.72 (1.33–5.60)	2.18 (1.04–4.58)	3.27 (1.62–6.62)	1.24 (1.08–1.43)	0.002
* Model 1*	1.00	1.87 (0.84–4.15)	2.18 (0.95–5.04)	3.31 (1.32–8.24)	1.31 (1.08–1.60)	0.007
***Non-cardia***						
* Cases/Controls*	48/194	84/194	118/194	103/195	352/777	
* Crude*	1.00	1.75 (1.17–2.63)	2.46 (1.66–3.63)	2.15 (1.44–3.19)	1.18 (1.08–1.28)	<0.001
* Model 1*	1.00	1.44 (0.86–2.39)	2.43 (1.44–4.09)	2.97 (1.64–5.39)	1.30 (1.14–1.50)	<0.001
***Histological subtype ^2^***						
***Diffuse***						
* Cases/Controls*	28/194	48/194	71/194	56/195	203/777	
* Crude*	1.00	1.71 (1.03–2.85)	2.53 (1.56–4.10)	2.00 (1.21–3.26)	1.16 (1.05–1.29)	0.005
* Model 1*	1.00	1.33 (0.73–2.43)	2.80 (1.54–5.10)	2.48 (1.23–5.00)	1.26 (1.08–1.48)	0.003
***Intestinal***						
* Cases/Controls*	23/194	46/194	54/194	51/195	174/777	
* Crude*	1.00	2.00 (1.17–3.43)	2.35 (1.38–3.98)	2.21 (1.30–3.75)	1.17 (1.05–1.31)	0.006
* Model 1*	1.00	1.66 (0.89–3.06)	2.52 (1.34–4.74)	2.82 (1.38–5.74)	1.30 (1.10–1.51)	0.002

^1^ Binary logistic regression. ^2^ Multinomial logistic regression. The mixed histological subtype was not considered due to the number of cases < 30. Reference: Quartile 1 (maximum anti-inflammatory diet). OR: Odds ratio. 95% CI: 95% confidence interval. Model 1: adjusted for sex, age (years), marital status, schooling, self-reported ethnicity, family history of cancer in first-degree relatives, study region, PPIs/H2RAs, antacids, NSAIDs, aspirin, nutritional supplement, BMI (categories), tobacco smoking, alcohol consumption, diabetes, sodium intake (g/day). ^a^ Hosmer–Lemeshow test = 11.00, *p* = 0.20 (E-DII quartiles) and 11.92, *p* = 0.16 (E-DII continuous). ‡ *p*-value for trend derived from models using the E-DII continuous variable.

## Data Availability

The datasets generated and/or analyzed during this study are available from the corresponding author upon request.

## Supplementary Materials

The following supporting information can be downloaded at: https://www.mdpi.com/article/10.3390/nu15132867/s1, Table S1: Sociodemographic, clinical, and nutritional characteristics of cases and controls by Energy-adjusted Dietary Inflammatory Index (E-DII) score quartiles.
