# Supplementary material for: Association between Dietary Inflammatory Index and Gastric Adenocarcinoma: A Multicenter Case-Control Study in Brazil
Source: Nutrients. 2023 Jun 24;15(13):2867. doi: 10.3390/nu15132867 (PMC10343640; doi:10.3390/nu15132867)
Supplement: Supplementary file 1 [file nutrients-15-02867-s001.zip › nutrients-2469272-supplementary.pdf]

Supplementary material

Table S1. Sociodemographic, clinical, and nutritional characteristics of cases and controls by Energy-adjusted Dietary Inflammatory Index (E-DII) score quartiles

	**E-DII quartiles §**														
	**Cases (*n*=492)**					**Control I (*n*=377)**					**Control II (*n*=776)**				
**Variables**	**Q1 n=123 (−4.35, −1.47)**	**Q2 n=124 (−1.46, −0.45)**	**Q3 n=122 (−0.44, 0.52)**	**Q4 n=123 (>0.53)**	***P*-value[1]‡**	**Q1 n=94 (−5.55, −2.01)**	**Q2 n=94 (−2.00, −0.74)**	**Q3 n=95 (−0.73, 0.56)**	**Q4 n=94 (>0.56)**	***P*-value[1]‡**	**Q1 n=194 (−5.09, −2.04)**	**Q2 n=194 (−2.03, −0.83)**	**Q3 n=194 (−0.82, 0.25)**	**Q4 n=194 (>0.25)**	***P*-value[1]‡**
	median (P_{25}, P_{75}) or *n* (%)														
Sex					0.01					0.23					<0.001
Female	66 (53.7)	49 (39.5)	42 (34.4)	42 (34.1)		57 (60.6)	45 (47.9)	45 (47.4)	48 (51.1)		119 (61.3)	99 (51.0)	80 (41.2)	80 (41.2)	
Male	57 (46.3)	75 (60.5)	80 (65.6)	81 (65.9)		37 (39.4)	49 (52.1)	50 (52.6)	46 (48.9)		75 (38.7)	95 (49.0)	114 (58.8)	114 (58.8)	
Age (years)	61 (51, 67)[a]	58 (49, 67)[ab]	57 (48, 64)[ab]	56 (45, 62)[c]	<0.001	60 (54, 68)[a]	56 (46, 63)[bc]	54 (43, 64)[bcd]	48 (37, 61)[d]	<0.001	56 (45, 67)[a]	59 (51, 66)[a]	53 (42, 64)[b]	49 (38, 60)[b]	<0.001
Self-reported ethnicity					0.01[2]					0.39[2]					<0.001
White	60 (48.8)	39 (31.5)	34 (27.9)	48 (39.0)		52 (55.3)	45 (47.9)	40 (42.1)	43 (45.7)		89 (45.9)	72 (37.1)	63 (32.6)	63 (32.5)	
Brown	50 (40.7)	67 (54.0)	71 (58.2)	61 (49.6)		28 (29.8)	33 (35.1)	37 (38.9)	36 (33.4)		64 (33.0)	76 (39.2)	93 (48.2)	87 (44.8)	
Black	5 (4.1)	13 (10.5)	13 (10.7)	13 (10.6)		8 (8.5)	9 (9.6)	16 (16.8)	10 (10.6)		19 (9.8)	23 (11.9)	30 (15.5)	38 (19.6)	
Others	8 (6.5)	5 (4.0)	4 (3.3)	1 (0.8)		6 (6.4)	7 (7.4)	2 (2.1)	5 (5.3)		22 (11.3)	23 (11.9)	7 (3.6)	6 (3.1)	
Schooling (years)					0.56					0.001					0.18
≤8	50 (40.7)	56 (45.2)	65 (53.3)	61 (49.6)		24 (25.5)	29 (30.9)	20 (21.1)	23 (24.5)		46 (23.7)	54 (27.8)	38 (19.6)	34 (17.5)	
9 a 12	43 (35.0)	43 (34.7)	36 (29.5)	37 (30.1)		28 (29.8)	36 (38.3)	57 (60.0)	42 (44.7)		95 (49.0)	95 (49.0)	108 (55.7)	114 (58.8)	
≥13	30 (24.4)	25 (20.2)	21 (17.2)	25 (20.3)		42 (44.7)	29 (30.9)	18 (18.9)	29 (30.9)		53 (27.3)	45 (23.2)	48 (24.7)	46 (23.7)	
Marital status					0.91					0.04					0.20
Married	91 (74.0)	89 (71.8)	88 (72.1)	85 (69.1)		64 (68.1)	64 (68.1)	64 (67.4)	55 (58.5)		136 (70.1)	134 (69.1)	130 (67.0)	123 (63.4)	

Single	17 (13.8)	15 (12.1)	19 (15.6)	20 (16.3)		9 (9.6)	20 (21.3)	13 (13.7)	24 (25.5)		35 (18.0)	27 (13.9)	38 (19.6)	47 (24.2)	
Others	15 (12.2)	20 (16.1)	15 (12.3)	18 (14.6)		21 (22.3)	10 (10.6)	18 (18.9)	15 (16.0)		23 (11.9)	33 (17.0)	26 (13.4)	24 (12.4)	
Family history of cancer in first-degree relatives					0.12					<0.001					0.06
No	38 (30.9)	56 (45.2)	44 (36.4)	50 (41.0)		24 (25.8)	45 (47.9)	51 (53.7)	39 (41.5)		84 (43.5)	94 (48.5)	108 (56.0)	105 (54.1)	
Yes	85 (69.1)	68 (54.8)	77 (63.6)	72 (59.0)		69 (74.2)	49 (52.1)	44 (46.3)	55 (58.5)		109 (56.5)	100 (51.5)	85 (44.0)	89 (45.9)	
Tobacco smoking					0.27					0.29					0.052
No	52 (42.3)	52 (42.3)	55 (45.5)	39 (32.0)		59 (64.1)	53 (56.4)	54 (56.8)	61 (64.9)		126 (65.3)	110 (57.0)	127 (66.1)	132 (68.0)	
Low	24 (19.5)	19 (15.4)	18 (14.9)	19 (15.6)		16 (17.4)	22 (23.4)	13 (13.7)	16 (17.0)		33 (17.1)	26 (13.5)	27 (14.1)	30 (10.3)	
Intermediate/High	47 (38.2)	52 (42.3)	48 (39.7)	64 (52.5)		17 (18.5)	19 (20.2)	28 (29.5)	17 (18.1)		34 (17.6)	57 (29.5)	38 (19.8)	42 (21.6)	
Alcohol consumption					0.056					<0.001					0.008
No	71 (57.1)	59 (47.6)	54 (46.2)	46 (37.4)		58 (64.4)	37 (39.8)	37 (39.4)	48 (51.1)		145 (75.9)	134 (69.8)	117 (61.6)	112 (59.3)	
Low	16 (13.0)	21 (16.9)	14 (12.0)	24 (19.5)		16 (17.8)	20 (21.5)	11 (11.7)	8 (8.5)		13 (6.8)	15 (7.8)	21 (11.1)	15 (7.9)	
Intermediate/High	36 (29.3)	44 (35.5)	49 (41.9)	53 (43.1)		16 (17.8)	36 (38.7)	46 (48.9)	38 (40.4)		33 (17.3)	43 (22.4)	52 (27.4)	62 (32.8)	
Diabetes					<0.001					0.38					0.11
No	97 (78.9)	117 (94.4)	115 (94.3)	118 (95.9)		81 (86.2)	83 (88.3)	78 (82.1)	85 (90.4)		176 (90.7)	176 (90.7)	183 (94.3)	186 (95.9)	
Yes	26 (21.1)	7 (5.6)	7 (5.7)	5 (4.1)		13 (13.8)	11 (11.7)	17 (17.9)	9 (9.6)		18 (9.3)	18 (9.3)	11 (5.7)	8 (4.1)	
Peptic ulcer					0.81					0.16[2]					-
No	115 (93.5)	113 (91.1)	110 (90.2)	113 (91.9)		91 (96.8)	85 (90.4)	92 (96.8)	91 (96.8)		-	-	-	-	
Yes	8 (6.5)	11 (8.9)	12 (9.8)	10 (8.1)		3 (3.2)	9 (9.6)	3 (3.2)	3 (3.2)		-	-	-	-	
H. pylori status					0.33					0.96					-
Negative	69 (84.1)	49 (72.1)	52 (75.4)	60 (76.9)		63 (71.6)	58 (69.9)	53 (67.9)	53 (70.7)		-	-	-	-	
Positive	13 (15.9)	19 (27.9)	17 (24.6)	18 (23.1)		25 (28.4)	25 (30.1)	25 (32.1)	22 (29.3)		-	-	-	-	
PPIs/H2RAs					0.22					0.44					0.049

	No	57 (47.1)	66 (53.2)	68 (56.2)	74 (60.2)		51 (54.8)	54 (57.4)	50 (52.6)	60 (63.8)		175 (90.7)	159 (82.4)	174 (89.7)	173 (89.2)	
	Yes	64 (52.9)	58 (46.8)	53 (43.8)	49 (39.8)		42 (45.2)	40 (42.6)	45 (47.4)	34 (36.2)		18 (9.3)	34 (17.6)	20 (10.3)	21 (10.8)	
Antacids						0.057					0.11					0.04[2]
	No	101 (82.1)	99 (79.8)	110 (90.9)	108 (87.8)		86 (92.5)	77 (81.9)	81 (85.3)	85 (90.4)		191 (99.0)	192 (99.0)	184 (94.8)	188 (96.9)	
	Yes	22 (17.9)	25 (20.2)	11 (9.1)	15 (12.2)		7 (7.5)	17 (18.1)	14 (14.7)	9 (9.6)		2 (1.0)	2 (1.0)	10 (5.2)	6 (3.1)	
Aspirin						0.02					0.13					0.009
	No	109 (88.6)	117 (94.4)	118 (97.5)	118 (95.9)		83 (89.2)	87 (92.6)	89 (93.7)	92 (97.9)		177 (91.7)	183 (94.3)	188 (96.9)	191 (98.5)	
	Yes	14 (11.4)	7 (5.6)	3 (2.5)	5 (4.1)		10 (10.8)	7 (7.4)	6 (6.3)	2 (2.1)		16 (8.3)	11 (5.7)	6 (3.1)	3 (1.5)	
Other NSAIDs						0.69					0.55					0.52
	No	106 (86.2)	110 (88.7)	107 (88.4)	112 (91.1)		79 (84.9)	85 (90.4)	82 (21.8)	85 (90.4)		181 (93.8)	188 (96.9)	183 (94.3)	184 (94.8)	
	Yes	17 (13.8)	14 (11.3)	14 (11.6)	11 (8.9)		14 (15.1)	9 (9.6)	13 (13.7)	9 (9.6)		12 (6.2)	6 (3.1)	11 (5.7)	10 (5.2)	
BMI (categories)						0.59					0.07					0.33
	Normal Weight	49 (39.8)	56 (45.2)	47 (38.5)	56 (45.5)		45 (48.4)	39 (41.5)	39 (41.1)	25 (26.6)		86 (44.3)	81 (42.0)	77 (39.7)	78 (40.2)	
	Underweight/Malnutrition	33 (26.8)	40 (32.3)	36 (29.5)	29 (23.6)		4 (4.3)	8 (8.5)	8 (8.4)	4 (4.3)		24 (12.4)	26 (13.5)	18 (9.3)	20 (10.3)	
	Overweight	19 (15.4)	17 (13.7)	22 (18.0)	20 (16.3)		23 (24.7)	25 (26.6)	29 (30.5)	41 (43.6)		49 (25.3)	44 (22.8)	67 (34.5)	62 (32.0)	
	Obese	22 (17.9)	11 (8.9)	17 (13.9)	18 (14.6)		21 (22.6)	22 (23.4)	19 (20.0)	24 (25.5)		35 (18.0)	42 (21.8)	32 (16.5)	34 (17.5)	
Energy intake (kcal/day)		2122 (1544, 2631)	2320 (1788, 2873)	2142 (1584, 2803)	2193 (1692, 2936)	0.11	1844 (1245, 2770)^{c}	1972 (1448, 2519)^{bc}	2144 (1642, 2828)^{ab}	2328 (1713, 3077)^{a}	<0.001	1729 (1362, 2310)	1813 (1460, 2405)	1837 (1242, 2474)	1832 (1360, 2392)	0.60
Sodium intake (g/day)		1.7 (1.4, 1.9)^{c}	1.7 (1.5, 2.1)^{abc}	1.8 (1.6, 2.3)^{abc}	6.2 (2.9, 8.1)^{a}	0.01	1.6 (1.3, 1.9)^{b}	1.7 (1.4, 2.0)^{ab}	1.8 (1.5, 2.1)^{a}	1.8 (1.6, 2.1)^{a}	<0.001	1.7 (1.4. 2.1)^{c}	1.8 (1.5, 2.1)^{c}	2.0 (1.6, 2.6)^{b}	4.8 (2.6, 7.7)^{a}	<0.001
Nutritional supplement						0.18					<0.001					<0.001
	No	94 (77.0)	103 (83.1)	104 (85.2)	107 (87.0)		68 (72.3)	68 (72.3)	88 (92.6)	84 (89.4)		152 (78.4)	164 (84.5)	178 (91.8)	177 (91.2)	
	Yes	28 (23.0)	21 (16.9)	18 (14.8)	16 (13.0)		26 (27.7)	26 (27.7)	7 (7.4)	10 (10.6)		42 (21.6)	30 (15.6)	16 (8.2)	17 (8.8)	

Numbers may differ because of missing values. Control I individuals (endoscopy); Control II individuals (hospital). PPIs/H2RAs: proton pump inhibitors/H2-receptor antagonists; NSAIDs: non-steroidal anti-inflammatory drugs; BMI: body mass index. § Based on cases, control I and control II scores. [1] Pearson χ^2 test or [2] Fisher's Exact tests for categorical variables. [1] Kruskal-Wallis test and Dunn-Bonferroni post hoc test for continuous variables. Different letters on the same line mean statistical difference between quartiles. ‡ *P*-value demonstrates difference between Quartile 4 (maximum pro-inflammatory diet) versus Quartile 1 (maximum anti-inflammatory diet) of the E-DII. Significance p-value < 0.05.
